# Global Integrated Genomic and Transcriptomic Analyses of MYB Transcription Factor Superfamily in C3 Model Plant *Oryza sativa* (L.) Unravel Potential Candidates Involved in Abiotic Stress Signaling

**DOI:** 10.3389/fgene.2022.946834

**Published:** 2022-07-08

**Authors:** Pandiyan Muthuramalingam, Rajendran Jeyasri, Anthonymuthu Selvaraj, Hyunsuk Shin, Jen-Tsung Chen, Lakkakula Satish, Qiang-Sheng Wu, Manikandan Ramesh

**Affiliations:** ^1^ Department of Biotechnology, Science Campus, Alagappa University, Karaikudi, India; ^2^ Department of Horticultural Science, Gyeongsang National University, Jinju, South Korea; ^3^ Department of GreenBio Science, Gyeongsang National University, Jinju, South Korea; ^4^ Department of Physiology and Biophysics, University of California, Irvine, Irvine, CA, United States; ^5^ Department of Life Sciences, National University of Kaohsiung, Kaohsiung, Taiwan; ^6^ Department of Biotechnology Engineering, Ben-Gurion University of the Negev, Beer Sheva, Israel; ^7^ College of Horticulture and Gardening, Yangtze University, Jingzhou, China

**Keywords:** abiotic stress, comparative mapping, MYB, Oryza sativa, phytohormone, qPCR, spatio-temporal, transcription factors

## Abstract

Plant transcription factors (TFs) are significant players in transcriptional regulations, signal transduction, and constitute an integral part of signaling networks. MYB TFs are major TF superfamilies that play pivotal roles in regulation of transcriptional reprogramming, physiological processes, and abiotic stress (AbS) responses. To explore the understanding of MYB TFs, genome and transcriptome-wide identification was performed in the C3 model plant, *Oryza sativa* (*OsMYB*). This study retrieved 114 *OsMYB* TFs that were computationally analyzed for their expression profiling, gene organization, *cis*-acting elements, and physicochemical properties. Based on the microarray datasets, six *OsMYB* genes which were sorted out and identified by a differential expression pattern were noted in various tissues. Systematic expression profiling of *OsMYB* TFs showed their meta-differential expression of different AbS treatments, spatio-temporal gene expression of various tissues and their growth in the field, and gene expression profiling in responses to phytohormones. In addition, the circular ideogram of *OsMYB* genes in related C4 grass plants conferred the gene synteny. Protein–protein interactions of these genes revealed the molecular crosstalk of *OsMYB* TFs. Transcriptional analysis (qPCR) of six *OsMYB* players in response to drought and salinity stress suggested the involvement in individual and combined AbS responses. To decipher how these *OsMYB* play functional roles in AbS dynamics, further research is a prerequisite.

## Introduction

Under environmental stress conditions, abiotic stresses (AbS) are the major players that negatively regulate the plant growth and thus crop yield. Generally, under field conditions, plants encounter combinations of two or more AbS (Bansal et al., 2012; Ahmad et al., 2019; Muthuramalingam et al., 2020a; Kayihan et al., 2021). To survive under natural field conditions, plants adapt and respond to combinations of these stresses through diverse biochemical, molecular, and physiological processes. The biological processes are controlled and/or regulated by transcriptional regulators which arbitrate the transcriptional and signaling regulation of several key players required for AbS tolerance. Therefore, delineating the regulatory mechanism of gene dynamism in response to various environmental cues is pivotal to developing the plant’s physiological processes for enhancing the agricultural yield (Hirayama and Shinozaki, 2010; Hadiarto and Tran, 2011; Sharoni et al., 2011; Sadiq et al., 2020).

Gene expression dynamism is controlled and/or regulated by various complex machineries, including transcriptional reprogramming, modifications to DNA such as DNA methylation and histone modification, and also diverse RNA-mediated processes. Transcription factors (TFs) are the key regulators of gene expression. In general, TFs have two different distinct domains, namely, 1) DNA binding and 2) transcriptional activator/repressor domains, ultimately directing the diverse cellular and biological processes through governing the transcriptional rates of AbS responsive target genes (Riechmann et al., 2000). Therefore, a better understanding of the transcriptional response of plants under AbS has remained the subject of comprehensive investigation for unraveling plant growth and developmental signature in the context of global climate change. TFs are regulatory proteins that can play a decisive role in accepting and converting the stress-induced signals to cellular responses that are essential for plants to be tolerant to AbS (Singh and Laxmi, 2015; Muthuramalingam et al., 2018a; Muthuramalingam et al., 2020a). In common, the plant genome contains ∼ 7% TFs and these are categorized into 58 TF families (Udvardi et al., 2007; Jin et al., 2014). Among TF families, MYB (myeloblastosis) comprises a significant amount (Udvardi et al., 2007).

The MYB family proteins are one of the largest TF families, functionally diverse in all eukaryotes and named by its conserved MYB domain. Plant MYB TFs are involved in controlling diverse molecular processes such as responses to abiotic and biotic stresses, differentiation, development, and defense metabolisms. They are generally classified into one to four direct repeats, namely, R1, R2R3, R3, and R4 family proteins (Du et al., 2009; Ambawat et al., 2013; Li et al., 2015). In plants, almost all the MYB proteins belong to the R2R3 family and play a predominant role in cell proliferation (Xie et al., 2010), cell cycle regulation (Cominelli and Tonelli, 2009), hormone signaling (Zhao et al., 2014), root system architecture (Dai et al., 2007), heat stress tolerance (Roy, 2016), diverse AbS responses (Seo et al., 2011; Katiyar et al., 2012; Zhang et al., 2012), several developmental processes (Komaki and Sugimoto, 2012), drought, salinity, low temperature, and UV (Smita et al., 2015; Roy, 2016; Wani S. H. et al., 2018). Various studies have shown the pivotal function of MYB TFs only in individual AbS, but not a combined AbS (CAbS). Therefore, understanding plant crosstalk to CAbS is essential and it needs to identify the CAbS-responsive candidate genes.

Taking this importance and lacunae into account, the current study aimed at high-throughput computational omics approaches to identify the potential AbS responsible (AbSR) for encoding MYB TF gene families from rice. Here, we applied transcriptome and hormonome-based analyses to dissect the MYB transcriptional regulatory network and their associated functions in rice. This is the first comprehensive study of MYB TFs in *O. sativa*. Thus, the study unravels novel avenues to identify MYB TF regulatory networks that direct the molecular biological processes and identify the candidates for further validation toward their functional role in CAbS dynamism.

## Materials and Methods

### Database Search, Identification, and Meta-Analysis of Rice MYB TFs

AbSR-MYB TFs and their protein sequences were retrieved from the GRASSIUS Grass Regulatory Information Server (Yilmaz et al., 2009). The MYB TFs and their respective RAP IDs/Gene locus IDs were collected. These IDs were then subjected to the Rice Oligonucleotide Array database (ROAD 2.0) meta-analysis expression search tool (Cao et al., 2012) to explore tissue-specific expression profiles in different tissues in rice and it was used for identifying the candidate genes. The AbSR-MYB TFs were used to collect the genomic transcript, coding sequences with their chromosomal localization from RiceSRTFDB (Priya and Jain, 2013).

### Gene Structure Analysis

The GSDS web server v2.0 was used to analyze the genomic and coding sequences of possible AbSR-MYB TFs in order to determine the role of introns and exons in the gene organization (http://gsds.cbi.pku.edu.cn/) (Hu et al., 2015).

### Spatio-Temporal and Phyto-Hormone Expression Profiling

RAP IDs of AbSR-MYB TFs were subjected to the RiceXPro database (http://ricexpro.dna.afrc.go.jp/) (Sato et al., 2010) which consists of the spatio-temporal and phyto-hormonal datasets (RXP_0001) and (RXP_1001∼RXP_1012), respectively, for analyzing the expression profiling of different tissues and plant hormone dynamisms of entire growth in the shoot and root under field conditions.

### *Cis*-Element Analysis

AbSR-MYB TF gene sequences were imported to plant *cis*-acting regulatory DNA elements (PLACE) online server (Higo et al., 1999) for predicting the *cis*-regulatory elements. All possible regulatory elements were matched in the 1 kb upstream region of AbSR-MYB TF in the online PLACE server. These elements are encoding the experimentally validated expression dynamism and strongly matched with rice promoters (Muthuramalingam et al., 2020b). The promoter signal sequence, factor site, position, and strand information were collected.

### Imputation of AbSR-MYB TFs Protein Properties and Subcellular Localization

The potential six AbSR-MYB TFs with their protein sequences were exposed to the ExPASy–ProtParam tool to impute the physicochemical features of MYB proteins (Gasteiger et al., 2005). The features include molecular weight (M.Wt), amino acid length (aaL), isoelectric point (pI), instability index (II), grand average of hydropathicity (GRAVY), and aliphatic index (AI). The subcellular localization of AbSR-MYB key proteins in *O. sativa* was predicted using CELLO2GO (Yu et al., 2014) and WoLF PSORT2 (Horten et al., 2007).

### Gene Ontology Enrichment Analysis

AbSR-MYB candidate genes with their respective locus IDs were uploaded to the Plant Transcriptional Regulatory Map (PlantRegMap) database to obtain GO annotation against *O. sativa* ssp. *japonica*. GO functional enrichment was calculated through Fisher’s exact test with a threshold *p*-value of 0.01 for the AbSR-MYB TFs. These players were classified based on their biological processes and molecular function in accordance with the PlantRegMap GO annotation.

### Protein–Protein Interaction Network Analysis

AbSR-MYB key players with their PPI network were conducted by STRING *v*11.0 (Szklarczyk et al., 2017) analysis with a high confidence score of 0.7 which is considered as a significant value. The physical position and molecular functional roles of the candidates involved were inferred using this interaction.

### Gene Synteny Analysis

AbSR-MYB TFs in rice with their orthologous genes in C4 panicoid genome grass plants such as *Setaria italica* (Si), *Zea mays* (Zm), and *Sorghum bicolor* (Sb) were predicted by reciprocal BLASTP analysis of amino acid sequences against these genomes. All the six MYB TFs sequences with a minimum of 80% homology were considered as significant. Chromosomal level synteny was then visualized by Circos *v*0.55 (Krzywinski et al., 2009).

### *In Silico* Expression Profiling of Key Players under AbS Treatments

Candidate MYB genes with their locus IDs were exposed to the Rice Oligonucleotide Array Database (ROAD 2.0) for analyzing the meta-differential expression profiling under various AbS treatments (Cao et al., 2012) in rice.

### Realtime PCR Analysis of Potential AbS Responsible *OsMYB* TF Players

To assess the expression dynamism of significant *OsMYB* TF players under drought and salinity stress conditions, qPCR analysis was performed. Primers (gene-specific) were designed for the 3′ UTR of each transcript using the GenScript® Real-time PCR (TaqMan) Primer Design tool and the details of the primer sequences for *OsMYB* TF players are given in Supplementary Table S1. The 14 days old *Oryza sativa* L. ssp. *japonica* cv. Nipponbare seedlings were used for stress treatments. Then, these seedlings were exposed to drought and salinity (250 mM NaCl) stress for 7 days and separate control was also maintained. Afterward, the total RNA was isolated from shoots (100 mg) of control, drought, and salinity-treated seedlings using the TRIzol method (TRI Reagent® -SIGMA ALDRICH). The samples were treated with RNase-free DNase I. Furthermore, cDNA conversion was performed by the High-Capacity cDNA Reverse Transcription Kit (Applied Biosystems Inc., United States). Transcriptional (qPCR) analysis was carried out in thermal cycler (7500 Sequence Detection System, Applied Biosystems Inc., Foster, CA, United States) using power SYBR green PCR master mix (Applied Biosystems Inc., United States) according to the manufactures’ protocol. The constitutive *Act2* gene was used as an endogenous control. The transcriptional expression level (2^−ΔΔ*CT*^) was calculated using comparative cycle threshold (Ct) value analysis with *Act2* gene normalization (Livak and Schmittgen, 2001; Muthuramalingam et al., 2021).

### Statistical Analysis

The experiments were conducted in three biological replicates with at least two technical replicates and data are presented as mean ± standard deviation (SD). The significant difference between the values of control and treated samples was analyzed through one-way analysis of variance (ANOVA) as well as Duncan’s *post hoc* test using the SPSS statistical software package *v*17.0 (Chicago, IL, United States). The significant *p*-value was fixed as <0.05.

## Results

### Identification of Potential MYB TFs and Their Attributes in Rice

A total of 114 retrieved rice MYB TFs were analyzed systematically for their tissue-specific expression profiling (Supplementary Figure S1). Of them, six MYB TFs are key candidates and responsible for individual and combined AbS, which have been showing higher expression signatures in diverse tissues (Supplementary Figure S2). This heatmap expression signature was also demonstrated, and its function was delineated in a tissue-specific manner. These potential AbSR-MYB protein/amino acid sequences were obtained from the GRASSIUS grass regulatory information server. The whole set of potential *OsMYB* TFs and their corresponding information such as locus IDs/RAP IDs were collected. Furthermore, genomic, coding, and protein sequences as well as chromosomal localization and UniProt ID were retrieved and presented in Table 1.

**TABLE 1:** Potential AbSR-*OsMYB* TFs and its details.

S.No	Gene name	Locus ID	RAP ID	Probeset ID	Gene position	Exon	Intron	Chromosome number	UniProt ID
1	*OsMYBR17*	LOC_Os01g64360	Os01g0863300	Os.49787.1.S1_at	37357161–37358553 (+)	2	1	1	Q0JHH7
2	*OsMYB50*	LOC_Os04g28090	Os04g0348300	Os.10202.1.S1_at	16407910–16415223 (+)	4	3	4	Q0JE02
3	*OsMYB55*	LOC_Os04g43680	Os04g0517100	Os.3388.1.S1_x_at	25657904–25659778 (−)	3	2	4	Q7XBH4
4	*OsMYB80*	LOC_Os06g40330	Os06g0605750	Os.22469.1.S1_at	24004102–24011311 (−)	7	6	6	Q69X09
5	*OsMYB81*	LOC_Os06g43090	Os06g0637500	Os.54569.1.S1_x_at	25898445–25899820 (−)	1	0	6	Q67WF6
6	*OsMYB102*	LOC_Os08g43550	-	Os.9336.1.S1_at	27545257–27546459 (−)	2	1	8	Q6ZJJ2

### Structural Analysis of AbSR-MYB TF Genes

**FIGURE 1:**
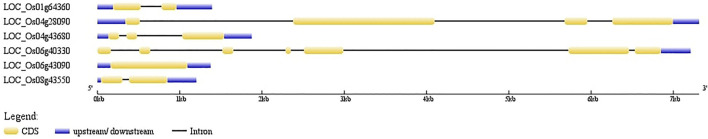
Candidate AbSR-*OsMYB* TF organization. The blue lines encode the UTR regions, orange boxes represent the exons, black line represents introns. Scale bar denotes the size of the gene.

Candidate AbSR-MYB TF genes and their organization of exons and introns were imputed. It showed the numbers and arrangements of exons and introns. Among the six MYB TFs, five MYB genes have introns, while one AbSR-MYB TF gene (*OsMYB81*; LOC_Os06g43090) was found to be intronless (Figure 1).

### Spatio-Temporal Expression Profiling of MYB TFs

**FIGURE 2:**
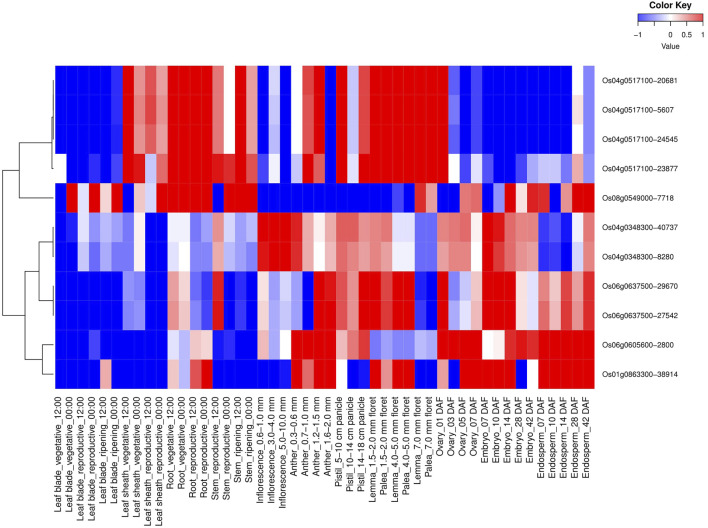
Heatmap depicts the spatio-temporal differential expression profiling of potential AbSR-*OsMYB* TFs in 48 different developmental tissues under natural field conditions. Red color—upregulation, blue color—downregulation; white color—unchanged. The colored scale bar at right side top denotes the relative expression value, where -1 and 1 represent down and upregulation of AbSR-*OsMYB* TFs, respectively. The colors also depict the individual expression values of tissues.

All 114 *OsMYB* TFs and their spatio-temporal expression profiling were analyzed (Supplementary Figure S3). Similar to tissue-specific expressions, six AbSR-MYB TF genes and their spatio-temporal expression profiling were also observed in 48 different developmental tissues at diverse developmental stages (Figure 2). These six MYB candidates showed higher expression levels in various developmental tissues/organs such as leaf blade (vegetative—00:00), leaf sheath (vegetative, reproductive—12:00, 00:00), root (vegetative, reproductive—12:00, 00:00), stem (reproductive, ripening—12:00, 00:00), anther (0.7–1.0, 1.2–1.5, and 1.6–2.0 mm), lemma (1.5–2.0, 4.0–5.0, and 7.0 floret), palea (1.5–2.0, 4.0–5.0, and 7.0 mm floret), ovary (01, 03, 05, and 07 DAF), embryo (07, 10, 14, 28, and 42 DAF), and endosperm (07, 10, 14, 28, and 42 DAF) (Figure 2). Furthermore, AbSR-MYB TFs exhibited low-level expressions in leaf blade (reproductive, ripening—12:00, 00:00), anther (0.3–0.6 mm), pistil (5–10, 14–18 cm panicle), and inflorescence (0.6–1.0, 3.0–4.0, and 5.0–10.0 mm) (Figure 2). The spatio-temporal transcriptomic results were analyzed by RiceXPro, based on the available AbSR-MYB TFs filed RNA-Seq data.

### Plant Hormonal Expression Dynamism of AbSR-MYB TFs

**FIGURE 3:**
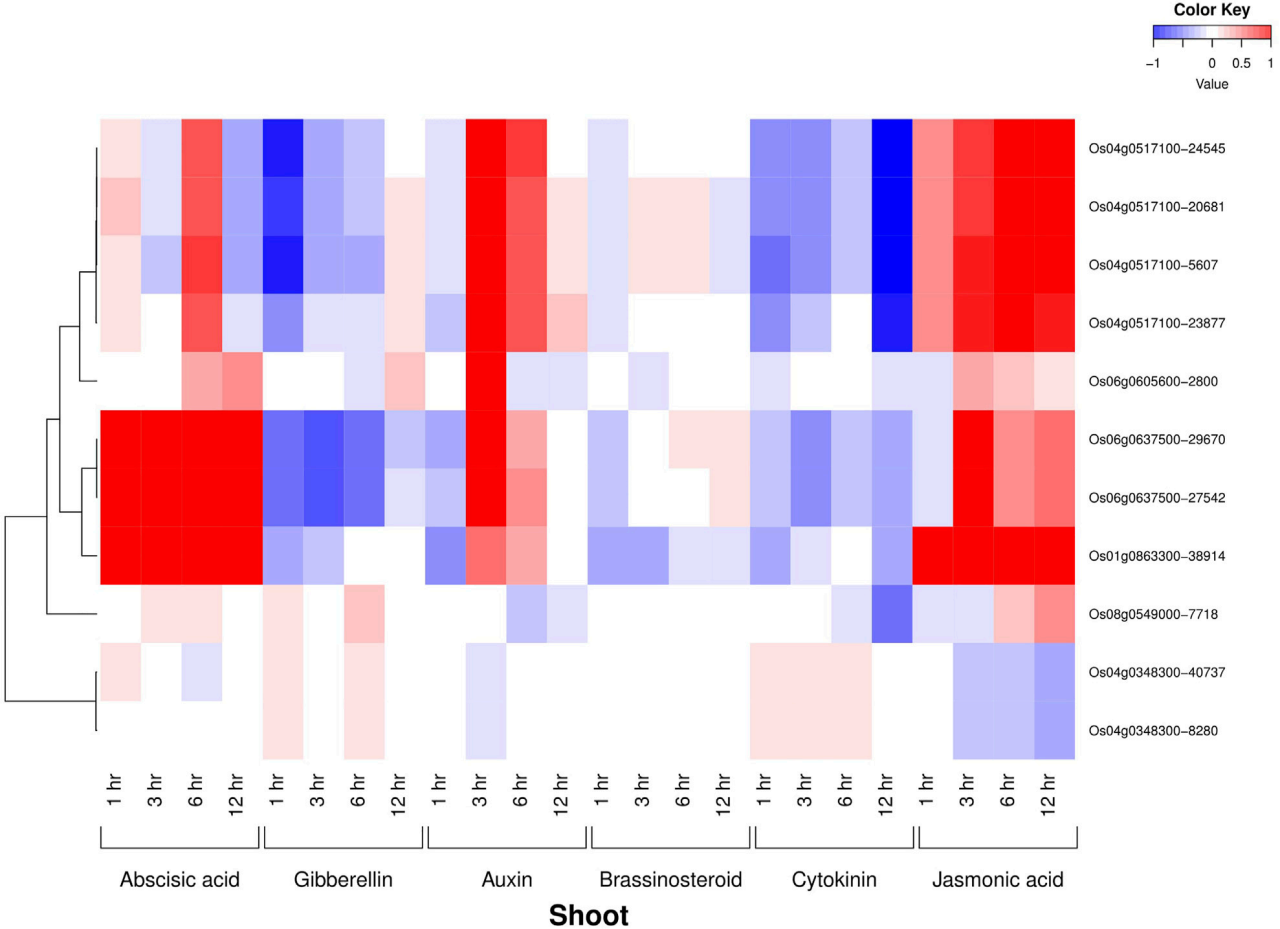
Heatmap denotes the six AbSR-*OsMYB* TFs and their plant hormonal expression signature in shoot across the whole growth under the field conditions. Red color—upregulation; blue color—downregulation; white color—unchanged. The colored scale bar at right side top indicates the relative expression value, where -1 and 1 represent down and upregulation of AbSR-*OsMYB* TFs, respectively. The colors also depict the individual expression values of shoot hormones.

**FIGURE 4:**
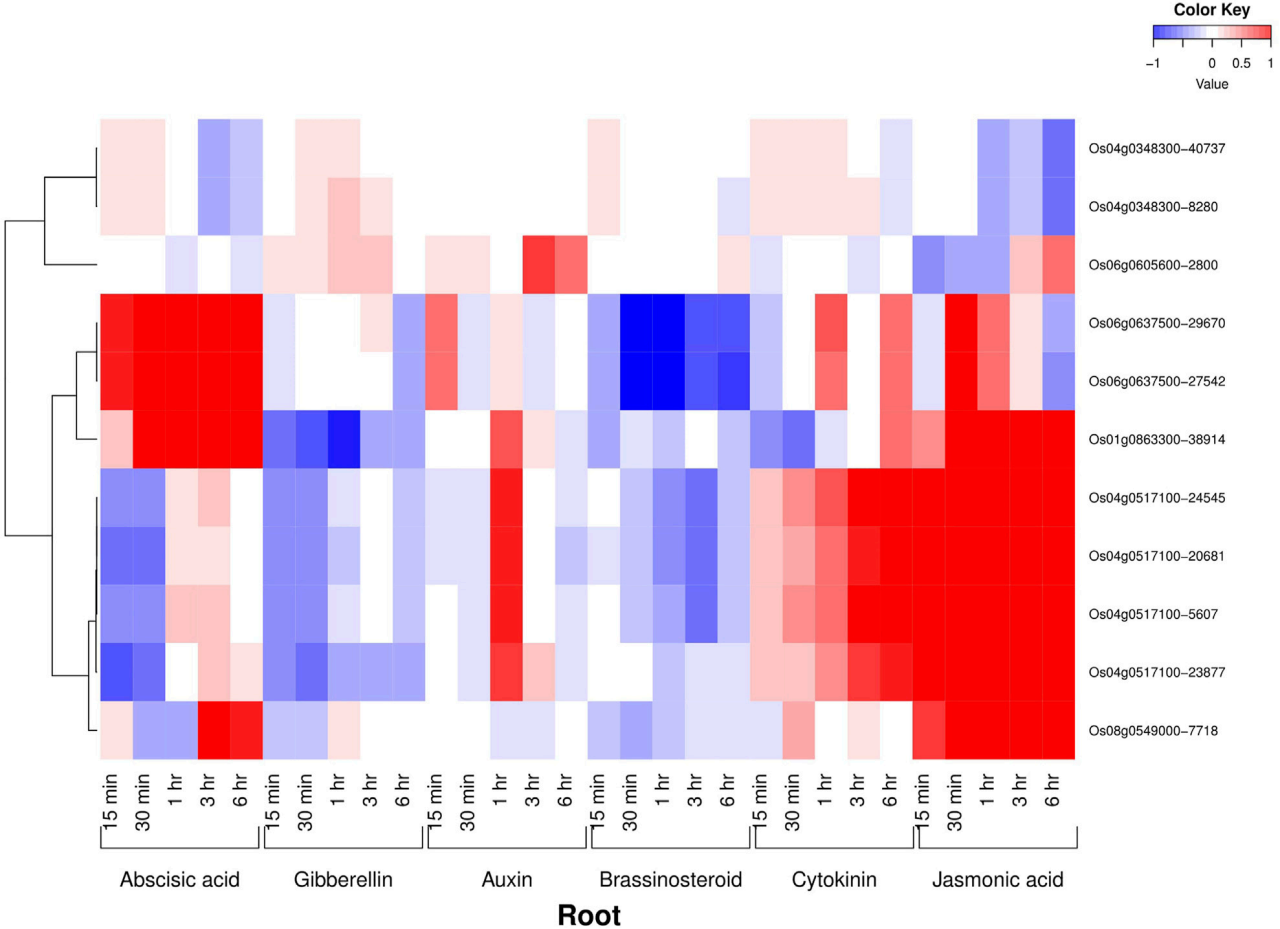
Heatmap denotes the six AbSR-*OsMYB* TFs and their hormonal expression patterns in root across the entire growth in the field. Red color—upregulation; blue color—downregulation; white color—unchanged. The colored scale bar at right side top indicates the relative expression value, where -1 and 1 represent down and upregulation of AbSR-*OsMYB* TFs, respectively. The colors also depict the individual expression values of root hormones.

All the six AbSR-MYB TFs exhibited their hormone expression signatures in diverse time points such as 1, 3, 6, 12 h and 15 min, 30 min, 1, 3, 6 h on the shoot and root, respectively, under environmental conditions and also in response to individual and CAbS conditions. In the shoot, an elevated level of expression was observed in abscisic acid (ABA), jasmonic acid (JA), and auxin (Aux) and a low-expression level was noticed in gibberellins (GAs), brassinosteroids (BRs), and cytokinins (CKs) at all the time points (Figure 3). In the root, the higher expression of ABA, JA, and CK and negligible expression of Aux, GAs, and BRs were observed in AbSR-MYB TFs in different time points (Figure 4).

### *Cis*-Regulatory Elements of AbSR-MYB TFs

Promoter imputations of significant six AbSR-MYB TF genes exhibited the presence of 1239 *cis*-acting regulatory elements. Among these elements, few elements were predominantly present in all six AbSR-MYB TFs, whereas few elements were present in one of two genes of AbSR-MYB TFs (Supplementary Table S2). EBOXBNNAPA (seed-specific storage protein, bHLH, MYB, E-box, and bZIP TF target binding site, ABRE (ABA-responsive element), MYB2CONSENSUSAT (response to drought, salinity, MYB TF binding site, expressed in leaf and seed, ABRE), MYCCONSENSUSAT (seed development, response to drought, cold, ABA (abscisic acid), MYC recognition site, ICE1 (inducer of CBF expression 1), and osmotic specific elements), DRECRTCOREAT (dehydration-responsive element/C-Repeats (DRE/CRT), response to drought, high-salt, high-light, CBF, DREB, and cold responsive gene expression), CBFHV (AP2 domain, low temperature DRE/CRT, CBF), SORLIP1AT (light, root, seed-specific element, and phytochrome A gene expression), WRKY71OS (WRKY and MYB TF binding site, gibberellin signaling pathway, W-box, and elicitor encoding elements), ARR1AT (cytokinin response regulator), and RHERPATEXPA7 (root hair–specific *cis*-elements) were imputed in the direct upstream region of potential six AbSR-MYB TFs (Supplementary Table S2). Moreover, some promoter elements were only present in one MYB TF which includes ABREOSRAB21 (ABA-responsive element) and DRE1COREZMRAB17 (late embryogenesis) present in *OsMYBR17*. ANAERO4CONSENSUS and ANAERO1CONSENSUS (response to anaerobic conditions) are present in *OsMYB50* and *OsMYB80*, respectively. SEBFCONSSTPR10A (defense response) and CPBCSPOR (chloroplast enhanced protein binding) are present in *OsMYB50*, QARBNEXTA (response to wounding in *OsMYB55*), and OCTAMOTIF2 (histone–gene–specific consensus sequences in OsMYB80). CAREOSREP1 and GAREAT (GA responsiveness) are present in *OsMYB81* and *OsMYB102*, respectively (Supplementary Table S2).

### Physicochemical Properties of Potentially Expressed MYB TFs

Among the six potential AbSR-MYB TFs with their encoding proteins, *OsMYB50* protein was the largest with 769 aaL and *OsMYB17* stand the smallest with 173 aaL. Pertaining to aaL, M. Wt also varied with *OsMYB50* being the biggest (87.67 kD) and *OsMYB17* (19.31 kD). These players were also predicted to have differences in pI ranging from 6.56 (*OsMYB55*) to 9.1 (*OsMYB102*). On the other hand, AI, II and GRAVY were also imputed and unanimously all the six proteins were found to be unstable (Table 2).

**TABLE 2:** Physicochemical features of MYB TFs.

S. No	Gene name	Locus ID	aaL	M.Wt	pI	II	AI	GRAVY	S/US
**MYB family**									
1	*OsMYBR17*	LOC_Os01g64360	173	19318.53	7.92	44.25	68.27	−0.782	US
2	*OsMYB50*	LOC_Os04g28090	769	87677.61	8.67	58.78	72.15	−0.924	US
3	*OsMYB55*	LOC_Os04g43680	257	27914.37	6.56	56.9	64.32	−0.591	US
4	*OsMYB80*	LOC_Os06g40330	682	75878.94	7.31	54.68	61.82	−0.771	US
5	*OsMYB81*	LOC_Os06g43090	311	33377.34	8.21	63.88	60.39	−0.539	US
6	*OsMYB102*	LOC_Os08g43550	243	26999.77	9.1	55.43	76.79	−0.554	US

aaL, amino acid length; M.Wt, molecular weight; pI, isoelectric point; II, instability index; AI, aliphatic index; GRAVY, grand average hydropathicity; S/US, stable/unstable.

### Subcellular Localization of Rice AbSR-MYB TFs

The subcellular localization search tools such as CELLO2GO and WoLF PSORT2 based on the high-resolution imputation principles and competences of these two programs. These six potential AbSR-MYB TFs in rice were predicted to be localized in the nucleus, mitochondria, and cytoplasm, respectively (Table 3). The result of this analysis revealed that the candidate MYB TFs are predominantly localized in the nucleus.

**TABLE 3:** Sub-cellular localization of AbSR-*OsMYB* TFs.

S. No	Gene name	Locus ID	WolfPsort	CELLO2GO
1	*OsMYBR17*	LOC_Os01g64360	Cyto	Cyto
2	*OsMYB50*	LOC_Os04g28090	Cyto	N
3	*OsMYB55*	LOC_Os04g43680	N	N
4	*OsMYB80*	LOC_Os06g40330	N	N
5	*OsMYB81*	LOC_Os06g43090	N	N
6	*OsMYB102*	LOC_Os08g43550	N	Mt

N, nucleus; Cyto, cytoplasm; Mt, mitochondria.

### Gene Ontology Functional Enrichment Analysis of AbSR-MYB TFs

**FIGURE 5:**
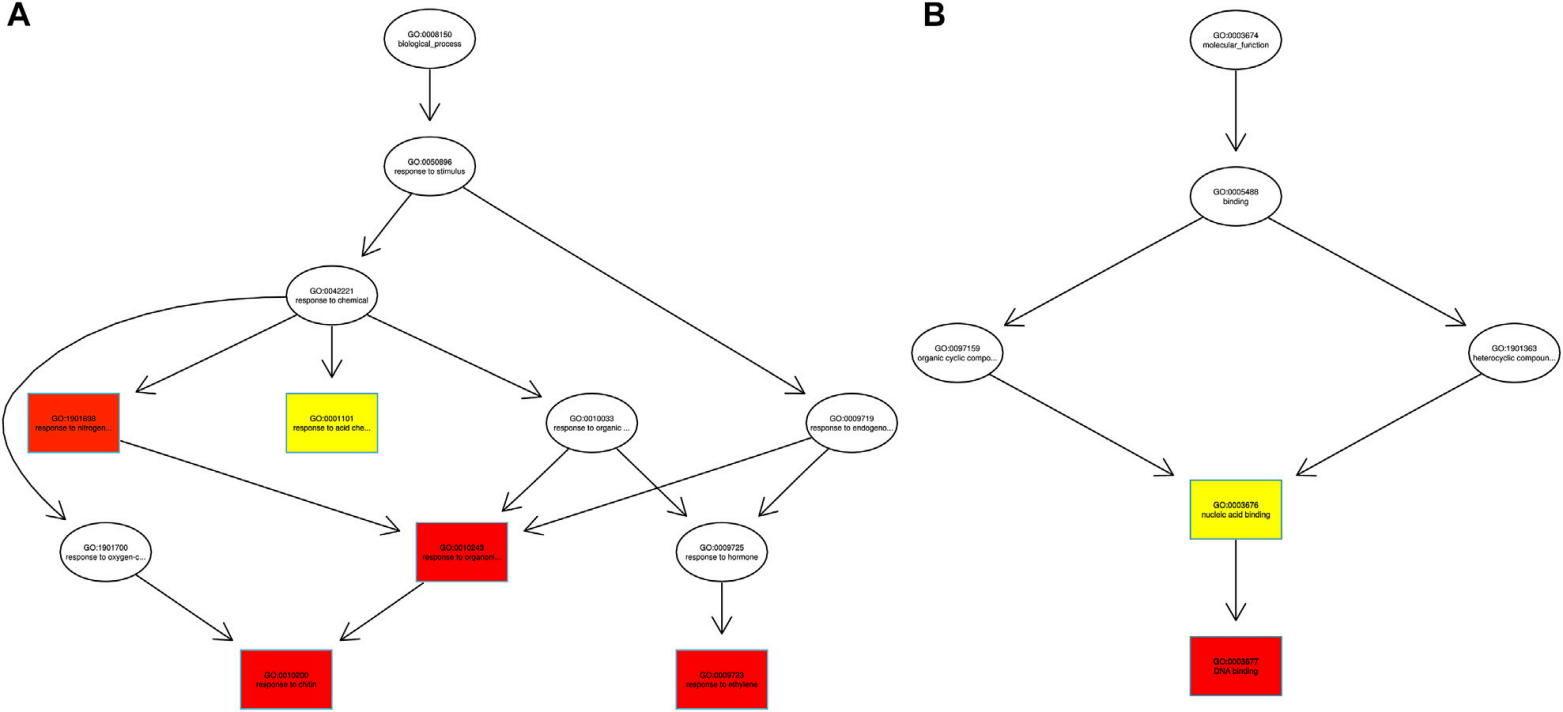
AbSR-*OsMYB* TFs with their GO enriched **(A)** biological processes and **(B)** molecular functions. The color filled boxes are according to the significance level (corrected *p*—value).

Fishers’ exact test was applied for functional GO annotation and found the enriched GO terms of the AbSR-MYB TFs. These candidate genes were putatively involved in diverse biological processes and molecular functions. Biological processes such as response to chitin, organo-nitrogen compound, ethylene, stress, and signal transduction are shown in Figure 5A. The molecular functions of these proteins correspond to different types of transcriptional regulator activity, DNA binding, and nucleic acid binding activity (Figure 5B).

### AbSR-MYB Gene Signaling Network

**FIGURE 6:**
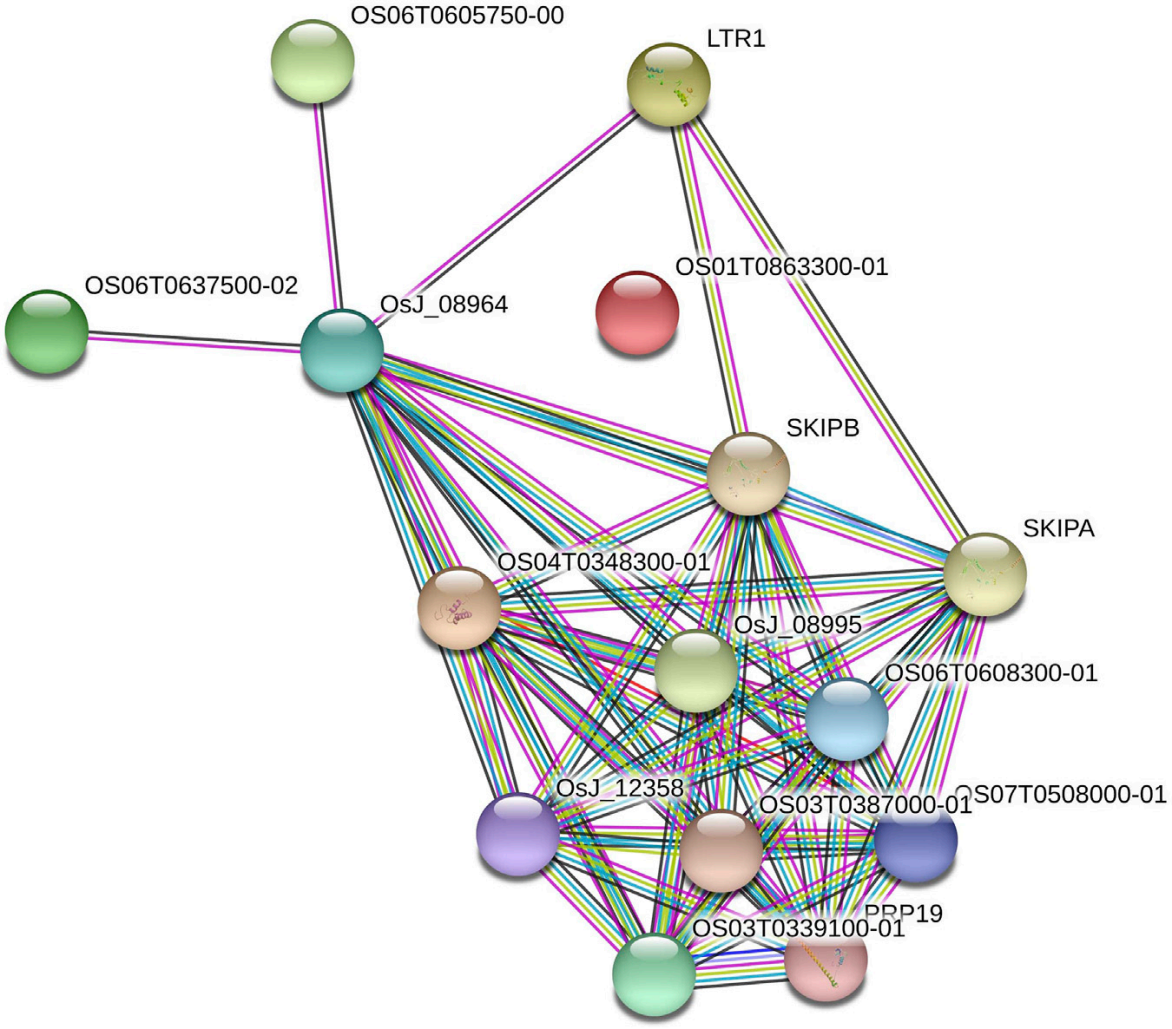
AbSR-*OsMYB* TFs with their PPI network. *Japonica* rice—AbSR-*OsMYB* TF interaction shows the tightly connected functional node. Colored line between the proteins denotes diverse types of interaction evidence. Enlarged protein nodes represent the availability of protein 3D structure information.

The six AbSR-MYB TFs encoding seed proteins were derived from *O. sativa* ssp*. japonica* AbSR-*OsMYB* gene interaction network analysis. The interaction network had 15 nodes and 60 edges (Figure 6). The seed proteins of AbSR-*OsMYB* crosstalk had an average node degree of eight and the local coefficient value is 0.887 than neighboring proteins. The complexity and biological insights of AbSR-*OsMYB* TFs were demonstrated in this molecular interaction network, exhibiting multigenic nature of these players. Moreover, this network also revealed that these key players have been involved in various pathways particularly signal transduction, hormone signaling, stress signal, and biosynthetic pathways.

### Comparative Mapping of AbSR-*OsMYB* TFs with C4 Grass Species

**FIGURE 7:**
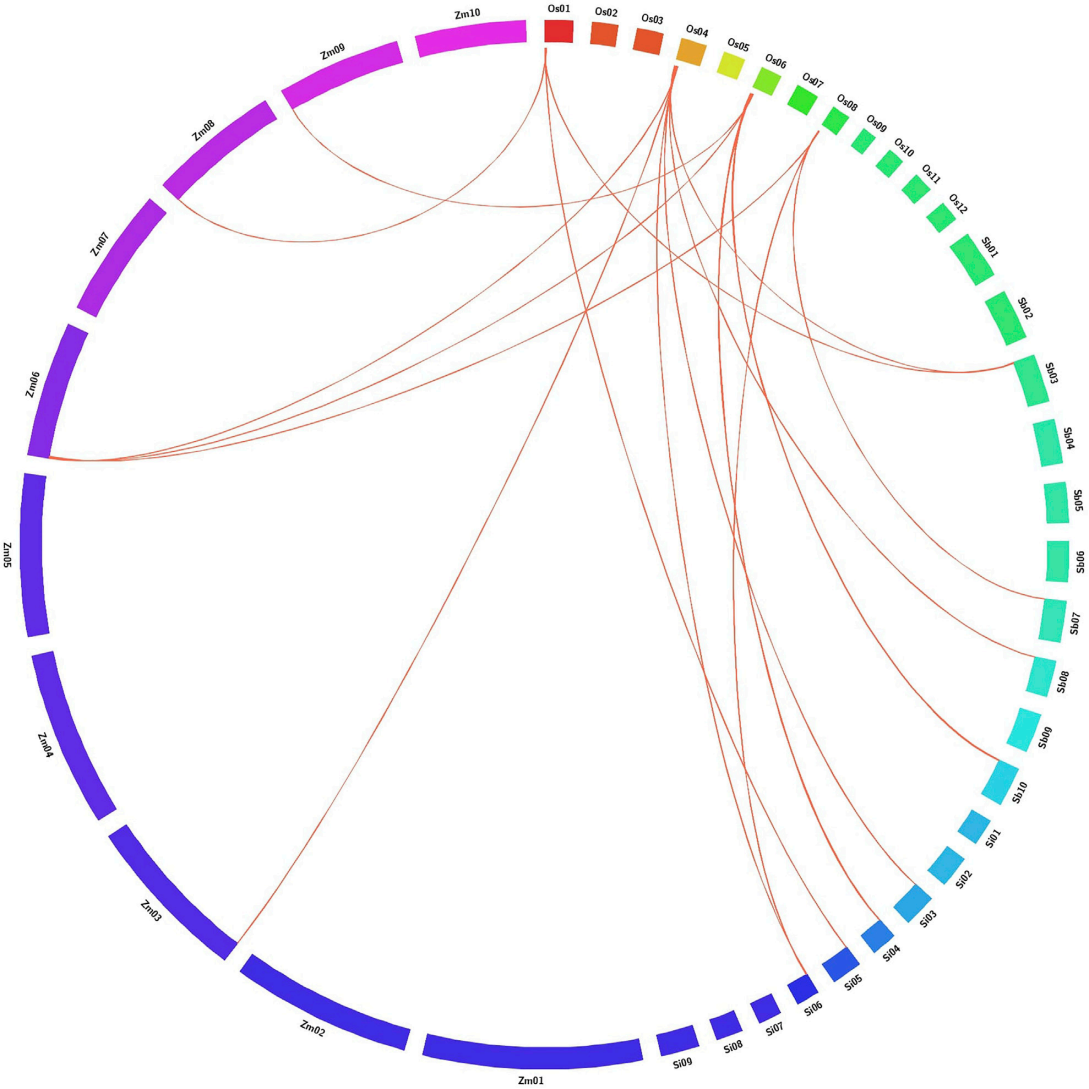
Comparative map shows the chromosomal collinearity of AbSR-*OsMYB* TFs in *O. s* *ativa* (Os), *S* *etaria italica* (Si), *Zea mays* (Zm), and *Sorghum bicolo* *r* (Sb). Each segment represents chromosome and the orthologous genomic regions are marked with red.

Reciprocal BLASTP analysis in Gramene exhibited significant gene synteny between six AbSR-*OsMYB* TFs and C4 panicoid genome grass plants such as *S. bicolor*, *Z. mays,* and *S. italica* based on the gene localization in difference with their corresponding chromosome. The result of comparative mapping revealed that rice and C4 grass species have the strongest maximum relationship [6 AbSR-*OsMYB* TFs (100%)] (Figure 7; Supplementary Table S3).

### Meta*-*Expression Profiling of Key Players under AbS Treatments

**FIGURE 8:**
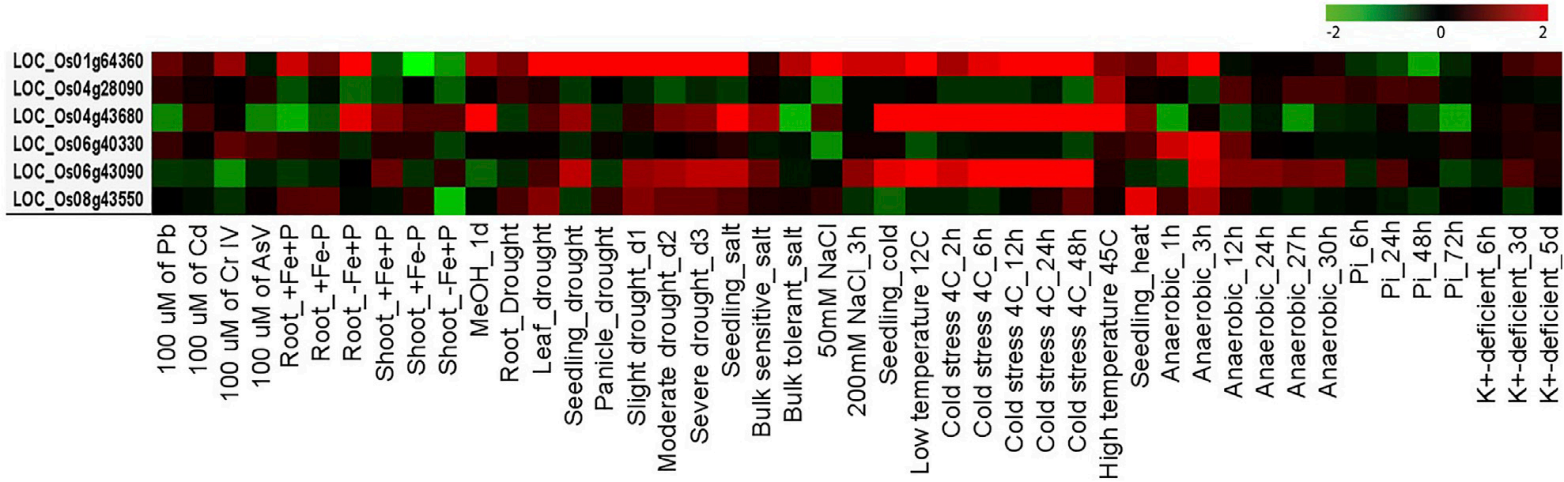
Differential expression patterns of rice MYB TFs. Heatmap represents expression profiling of AbSR-*OsMYB* TFs with respect to various AbS treatment conditions. Red color—upregulation; green color—downregulation; black color—unchanged. The colored scale bar at right side denotes the relative expression value, where -2 and 2 represent downregulation and upregulation, respectively. The colors also depict the individual expression values of abiotic stressors.

AbSR-*OsMYB* TF key players and their meta-AbS expression signatures were shown under 45 different AbS treatment conditions (Figure 8). Of them, three genes *OsMYBR17*, *OsMYB55*, and *OsMYB81* are predominantly expressed under various AbS treatment conditions such as Root_-Fe-P, drought (leaf, seedling, and panicle), slight, moderate, severe (drought_d1, d2, and d3), seedling (salt, cold), 50 mM NaCl, low temperature 12C, cold stress (4C_2, 6, 12, 24, and 48h), and high temperature 45C. All other three *OsMYB* TFs showed negligible expression level under the remaining AbS treatment conditions (Figure 8). The meta-AbS differential expression analysis results were analyzed by ROAD 2.0, based on the available AbSR-*OsMYB* TFs under microarray data of AbS treatments.

### Expression Profiling of *OsMYB* TF Genes in Response to Abiotic Stresses

**FIGURE 9:**
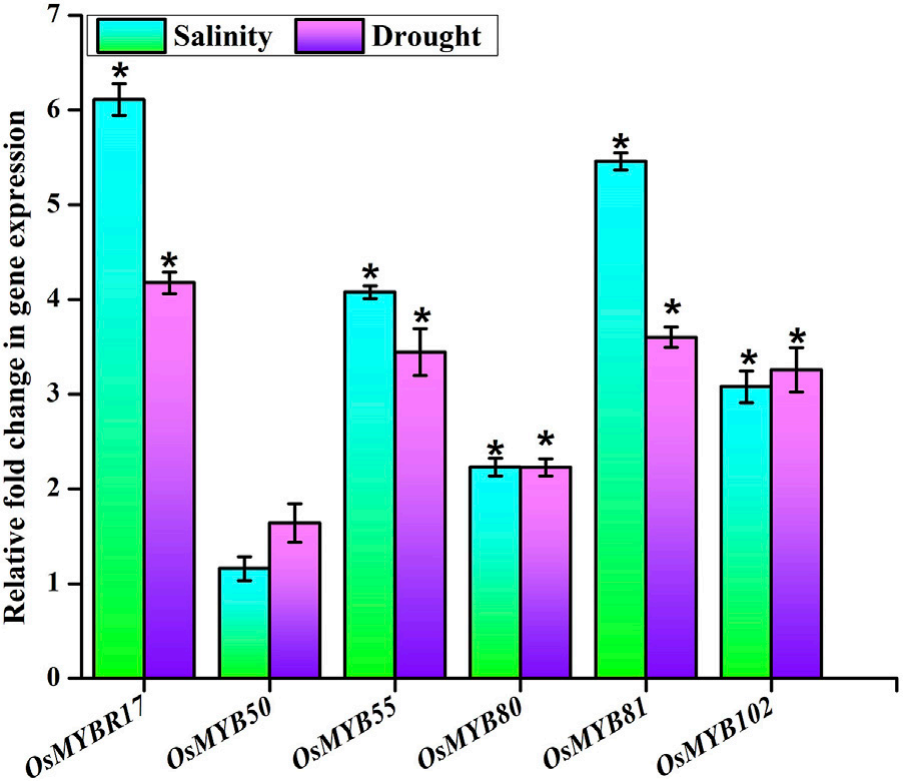
Transcriptional analysis. Expression level of six *OsMYB* TF players in seedlings of rice treated with drought and salinity stresses showed as a bar diagram. Endogenous control (*Act2* gene) was used to normalize the data. The error bars and asterisks symbols represent the SD and statistical significance (*p ≤ 0.05*), respectively.

The transcriptional expression signatures of six *OsMYB* TFs were assessed during salinity and drought treatments. The expression abundance determined using qPCR analysis showed a differential expression pattern of the *OsMYB* TF players involved (Figure 9). The results revealed the significant upregulation of the expression of candidate genes such as *OsMYBR17* (LOC_Os01g64360), *OsMYB50* (LOC_Os04g28090), *OsMYB55* (LOC_Os04g43680), *OsMYB80* (LOC_Os06g40330), *OsMYB81* (LOC_Os06g43090), and *OsMYB102* (LOC_Os08g43550) and represented their roles for further functional characterization and validation.

## Discussion

The effects of unique and CAbS factors (namely, drought, salinity, cold, high temperature, submergence, and UV and metal stresses) are the major bottleneck for plant growth, development, and productivity (Grennan, 2006; Mittler, 2006; Mittler and Blumwald, 2010; Breviario and Genga, 2013; Muthuramalingam et al., 2017; Pandey et al., 2017; Muthuramalingam et al., 2018a; Wani W. et al., 2018). The MYB TFs have been involved in various developmental processes, meristem formation, AbS, and physiological responses such as plant development, environmental stimuli, cell fate and identity, cell differentiation, proliferation, anatomical structure development, phenylpropanoid biosynthesis, determination of cell shape, floral and seed development, cell cycle control, and plant defenses (Noda et al., 1994; Abe et al., 2003; Araki et al., 2004; Hichri et al., 2011; Katiyar et al., 2012; Ambawat et al., 2013; Yang et al., 2014; Smita et al., 2015; Deeba et al., 2017). This class of TFs is one of the important proteins with their molecular crosstalk/signaling, involved in several pathways, auto-regulation, and plant-specific responses including primary and secondary metabolism which have already been reported (Dubos et al., 2010; Akhtar et al., 2012; Smita et al., 2015; Zhang et al., 2015).

The expression of MYB TFs in response to various AbS encodes their putative associations in the regulation of transcriptional reprogramming, gene expression, and accepts and transduces the various signals under environmental stress. Unveiling the systems insights of *OsMYB* TFs will be an essential source for us to develop the crop with enhanced nutrients and tolerance against AbS, especially on CAbS hurdles. Considering this importance, genome-wide analysis of the MYB TF family was reported previously in various model plants including rice (Baldoni et al., 2015; Roy, 2016). Hence, spatio-temporal, hormonal, different AbS treatment, differential expressional analysis of *OsMYB* TFs, molecular interactome, physiochemical features, subcellular localization, and chromosomal dating analysis are not reported so far in *Oryzoideae* C3 model crop plant rice with significant tolerance to unique and CAbS. These findings will open the floodgates for plant stress tolerance and avoidance control.

The aim of the pilot study was to investigate the unique and CAbS responsible for *OsMYB* TFs analysis in rice and also in C4 panicoid genome grass plants. Considering the active nature of its important functions in AbS biology in rice, this foremost study identified and retrieved about 114 MYB TF family members. All the rice MYB TF family members are involved in plant growth, development, and various stress responses at the minimum level. However, according to ROAD 2.0 (tissues) and RiceXPro (spatio-temporal) heatmap profiling, highly expressed six MYB AbSR TF players (*OsMYBR17*, *OsMYB50*, *OsMYB55*, *OsMYB80*, *OsMYB81*, and *OsMYB102*) were obtained and those were involved in individual and CAbS.

Analyzing the gene organization of six potent AbSR-*OsMYB* candidates revealed the variations in the transcript sequence and found one intronless gene which represents that the evolutionary changes have occurred in the rice genome (International Rice Genome Sequencing Project, 2005). In addition, intronless genes are significant gene families involved in various biological pathways such as amino acid biosynthesis, energy metabolisms, DNA binding, signal transduction, and translation and they may act as housekeeping genes, cellular processes, plant growth, and development (Muthuramalingam et al., 2021). Furthermore, the gene structure analysis throws light on the functional significance of six *OsMYB* TF genes in AbS mechanisms in rice and it enables the way for investigating the genesis of the genes.

Spatio-temporal expression analysis of six AbSR-*OsMYB* TFs showed the differential expression pattern in 48 different developmental tissues/organs and conferred the predominant level of expression dynamism under natural field conditions. In addition, tissue-specific (spatio-temporal) expression signature revealed the baseline knowledge of molecular and tissue-specific regulations under the field environment conditions (Sato et al., 2013; Muthuramalingam et al., 2020a). In addition, the key players open-up the novel avenues for conducting genetic and system analysis as well as metabolic engineering studies, by unraveling the biosynthetic pathways with their expression in various tissues to improve the AbSR TFs in rice.

Plant hormonal analysis revealed that the differential expression dynamism of six AbSR-*OsMYB* TFs in response to plant hormones such as ABA, Aux, CKs, GAs, BRs, and JA in shoots and roots of the rice plant throughout the entire growth in the field at various time points. Furthermore, the obtained results state that under field conditions, hormonal levels of ABA, Aux, CKs, and JA are low. However, elevated levels of hormonal expression of these plant hormones were discovered, indicating that these primary candidates can also play a role under stress conditions (Sato et al., 2013; Muthuramalingam et al., 2020a). Hence, hormone profiling brought new insights into delineating the expression profile of the AbSR-*OsMYB* TFs under AbS and field conditions.

Understanding the in-depth regulatory functions of potential AbSR-MYB TF that played a major role in various molecular physiological and stress responses via screening the *cis*-elements against the PLACE server. Significantly, we found plant developmental and AbSR *cis*-regulatory elements in the 1 kb upstream region of the transcription start site (TSS)/start codon (ATG) of the potential AbSR-MYB TF. Various numbers of cis-acting regulatory elements are directly associated to various TFs (ABRE, MYB, bZIP, WRKY, MYC, and bHLH) (Narusaka et al., 2003; Liang and Jiang, 2017; Muthuramalingam et al., 2018a) and abiotic stresses (drought, salinity, cold, temperature, and osmotic stresses) (Yamaguchi-Shinozaki and Shinozaki, 2005; Hou et al., 2012; Hernandez-Garcia and Finer, 2014; Muthuramalingam et al., 2018b).

The AbSR-*OsMYB* TFs with their PPI network revealed that the complex cross-talks of AbS among closely related proteins and their connecting nodules, edges, and candidates were connected and/or expressed in unique and CAbS (Muthuramalingam et al., 2018b; Muthuramalingam et al., 2020a). All the candidates of *OsMYB* TFs and their encoding proteins have variance in aaL, M. Wt, pI AI, II, and GRAVY values of these essential proteins. In addition, subcellular localization of these players at individual organelles is required to decode the existence of novel variants and more translational research to define their molecular physiological functions and their comprehensive picture of pathways.

The orthologous relationship of AbSR-*OsMYB* TFs with their respective genes on *O. sativa*, *S. italica*, *Z. mays*, and *S. bicolor* were collected and imputed to reveal the gene dating between the C4 grass plants. Candidate *OsMYB* TFs showed maximum collinearity with *SiMYB*, *SbMYB*, and *ZmMYB* (∼100%) owing to its broad-spectrum orthologous collinearity. These relationships showed evolutionary insights about C3 and C4 crop plant species. Moreover, a close gene synteny pattern was also observed in the comparative mapping of *OsHSF* TFs and AbSR-ThrMPG (Muthuramalingam et al., 2018b; Muthuramalingam et al., 2020a). On the whole, the Circos mapping could pave the way to analyze the evolutionary process of the AbSR-*OsMYB* TFs and to conduct breeding studies among the Gramineae family members.

Meta-differential expression pattern of six AbSR-*OsMYB* TFs exhibited the differential expression pattern under 45 different AbS treatment conditions and revealed a higher expression level under AbS treatments. The expression profiling of these treatment results strongly suggested that the identified six AbSR-*OsMYB* are playing a major role in unique and CAbS conditions, diverse biological processes, and molecular interactions. In addition, El-kereamy et al. (2012) reported that the overexpression of *OsMYB55* TF elevates the tolerance and immunity in rice plants to high temperature and also improved the amino acid metabolisms, especially glutamic acid and arginine metabolisms via transcriptional activation. These results are being used to pave the new avenues for identifying crucial players and conducting translational research in rice and other crop plants. Furthermore, Nakashima et al. (2014) states that TFs are essential candidates for overexpression or engineering the stress tolerant plants as single TF can modulate/cascade the large set of genes, especially downstream genes. The study leads to the development of plants with enhanced stress tolerance under field conditions with less negative impact on crop production.

The qPCR analysis of candidate players exhibited their differential expression patterns during salinity and drought stress treatments and revealed the putative involvement of the potential AbSR-*OsMYB* TF genes in AbS response and their role in hormonal regulation. Since rice is susceptible to AbS such as drought, salinity, cold, flood, and temperature (Muthuramalingam et al., 2017; Jeyasri et al., 2021), decoding the role of *OsMYB* TFs will offer the new insights into the stress tolerance mechanism of this crop. Altogether, the qPCR results unveiled that all the six AbSR-*OsMYB* TFs in response to drought and salinity stress would serve as an essential foundation for downstream gene characterization of these TFs.

## Conclusion

MYB is one of the major TF superfamilies in plants and it plays a significant role in signal transduction, stress responses, plant growth and development, and is involved in diverse biosynthetic pathways. The present study unveils AbSR-MYB TFs from rice and C4 panicoid genome grass plants such as maize, foxtail millet, and sorghum. *In silico* omics analyses of key candidates and their respective physicochemical features, gene structure, subcellular localization, and *cis*-regulatory elements revealed the putative roles of these players. Spatio-temporal and plant hormonal expression signatures delineate the molecular biological and biochemical insights of these candidates. Moreover, chromosomal dating of these genes exhibited that maximum homology with maize, foxtail millet, and sorghum. Generally, AbSR-*OsMYB* TFs regulate the MYB TF–associated genes, biological processes such as environmental and endogenous stimulus, regulation of two-component signal transduction and growth under AbS. *In silico* meta-analysis expression profiling revealed that these AbSR-*OsMYB* genes were differentially expressed under various AbS treatment conditions. In addition, transcriptional expression analysis of these major players under drought and salinity treatments exhibited the transcriptional expression signature of these genes, indicating that they are functionally regulated in individual AbS signaling. Overall, these six AbSR-*OsMYB* TFs can induce the various biosynthetic regulations, unique and CAbS tolerance, and play an essential role in enhancing the production and increasing the life span of the model plants. More studies are needed to delineate the novel avenues.

The present study also hypothesizes that AbSR-*OsMYB* candidates and their encoding proteins may interact with many other TFs as well as activate the many genes which were present in the downstream region. These candidates could induce the regulation of osmolytes and they play a significant role in AbS resistance and avoidance mechanisms. Furthermore, AbSR-*OsMYB* TFs frequently provide AbS endurance by altering the transcriptional and signal transduction processes in plants. The *in silico* expression analyses are based on the available expression (microarray) datasets in public repositories from previously conducted experiments in rice to delineate the transcriptional regulation of AbSR-*OsMYB* TFs in signal transduction and AbS dynamism. These analyses were used to understand the role of stress-related pathways and inhibit the activation and regulation of ROS-associated players. Hence, new technologies are a prerequisite to unveiling the comprehensive mechanism of AbSR-*OsMYB* TFs under individual and CAbS. In addition, combinatorial omics approaches, whole plant tissue type modeling, systems and synthetic biology, and genomic imprinting/epigenetics will be used to unravel the novel avenues of plant growth, development, and AbS functional and regulatory mechanisms. It will be the main base for conducting gene editing and genetic engineering studies. Furthermore, a possible extension of the current study using food cereals as well as model plants is speculated to throw more light on the *OsMYB* candidates’ role and their enhanced mode of action of AbS tolerance.

## Data Availability

The original contributions presented in the study are included in the article/Supplementary Material; further inquiries can be directed to the corresponding authors.

## Supplementary Material

Supplementary Figure S1 Differential expression patterns of rice MYB TF family genes. Heatmap represents expression profiling of MYB TF genes with respect to various specific tissues. Yellow color—upregulation; blue color—downregulation; black color—unchanged expression. The colored scale bar at right side denotes relative expression value, where 5.0 and 13.0 represent downregulation and upregulation, respectively.

Supplementary Figure S2 Heatmap profiling of potential AbSR-OsMYB TF family genes. Yellow color—upregulation; blue color—downregulation; black color—unchanged expression. The colored scale bar at right side denotes the relative expression value, where 5.0 and 13.0 represent downregulation and upregulation, respectively.

Supplementary Figure S3 Heatmap depicts the spatio-temporal differential expression profiling of 114 OsMYB TFs in 48 different developmental tissues under field conditions. Red color—upregulation; blue color—downregulation; white color—unchanged. The colored scale bar at the right side top denotes the relative expression value, where −1 and 1 represent down and upregulation of OsMYB TFs, respectively. The colors also depict the individual expression values of tissues.

## References

[B1] Abe H. Urao T. Ito T. Seki M. Shinozaki K. Yamaguchi-Shinozaki K. (2003). Arabidopsis AtMYC2 (bHLH) and AtMYB2 (MYB) Function as Transcriptional Activators in Abscisic Acid Signaling. Plant Cell 15, 63–78. 10.1105/tpc.006130 12509522 PMC143451

[B2] Ahmad B. Zaid A. Sadiq Y. Bashir S. Wani S. H. (2019). “Role of Selective Exogenous Elicitors in Plant Responses to Abiotic Stress Tolerance,” in Plant Abiotic Stress Tolerance. Editors Hasanuzzaman M. Hakeem K. Nahar K. Alharby H., 273–290. 10.1007/978-3-030-06118-0_12

[B3] Akhtar M. Jaiswal A. Taj G. Jaiswal J. P. Qureshi M. I. Singh N. K. (2012). DREB1/CBF Transcription Factors: Their Structure, Function and Role in Abiotic Stress Tolerance in Plants. J. Genet. 91, 385–395. 10.1007/s12041-012-0201-3 23271026

[B4] Ambawat S. Sharma P. Yadav N. R. Yadav R. C. (2013). MYB Transcription Factor Genes as Regulators for Plant Responses: an Overview. Physiol. Mol. Biol. Plants 19 (3), 307–321. 10.1007/s12298-013-0179-1 24431500 PMC3715649

[B5] Araki S. Ito M. Soyano T. Nishihama R. Machida Y. (2004). Mitotic Cyclins Stimulate the Activity of C-myb-like Factors for Transactivation of G2/M Phase-specific Genes in Tobacco. J. Biol. Chem. 279, 32979–32988. 10.1074/jbc.m403171200 15175336

[B6] Baldoni E. Genga A. Cominelli E. (2015). Plant MYB Transcription Factors: Their Role in Drought Response Mechanisms. Ijms 16 (7), 15811–15851. 10.3390/ijms160715811 26184177 PMC4519927

[B7] Bansal K. C. Katiyar A. Smita S. Chinnusamy V. (2012). Functional Genomics and Computational Biology Tools for Gene Discovery for Abiotic Stress Tolerance. Improv. Crop Resist. Abiotic Stress, 321–335. 10.1002/9783527632930.ch14

[B8] Breviario D. Genga A. (2013). Stress Response in Rice. J. Rice Res. 2. 10.4172/jrr.1000e104

[B9] Cao P. Jung K.-H. Choi D. Hwang D. Zhu J. Ronald P. C. (2012). The Rice Oligonucleotide Array Database: an Atlas of Rice Gene Expression. Rice 5 (1), 17. 10.1186/1939-8433-5-17 24279809 PMC4883718

[B10] Cominelli E. Tonelli C. (2009). A New Role for Plant R2R3-MYB Transcription Factors in Cell Cycle Regulation. Cell Res. 19 (11), 1231–1232. 10.1038/cr.2009.123 19881525

[B11] Dai X. Xu Y. Ma Q. Xu W. Wang T. Xue Y. (2007). Overexpression of an R1R2R3 MYB Gene, OsMYB3R-2, Increases Tolerance to Freezing, Drought, and Salt Stress in Transgenic *Arabidopsis* . Plant Physiol. 143 (4), 1739–1751. 10.1104/pp.106.094532 17293435 PMC1851822

[B12] Deeba F. Sultana T. Javaid B. Mahmood T. Naqvi S. M. S. (2017). Molecular Characterization of a MYB Protein from Oryza Sativa for its Role in Abiotic Stress Tolerance. Braz. Arch. Biol. Technol. 60. 10.1590/1678-4324-2017160352

[B13] Du H. Zhang L. Liu L. Tang X.-F. Yang W.-J. Wu Y.-M. (2009). Biochemical and Molecular Characterization of Plant MYB Transcription Factor Family. Biochem. Mosc. 74 (1), 1–11. 10.1134/s0006297909010015 19232042

[B14] Dubos C. Stracke R. Grotewold E. Weisshaar B. Martin C. Lepiniec L. (2010). MYB Transcription Factors in Arabidopsis. Trends Plant Sci. 15, 573–581. 10.1016/j.tplants.2010.06.005 20674465

[B15] El-kereamy A. Bi Y.-M. Ranathunge K. Beatty P. H. Good A. G. Rothstein S. J. (2012). The Rice R2R3-MYB Transcription Factor OsMYB55 Is Involved in the Tolerance to High Temperature and Modulates Amino Acid Metabolism. PLoS One 7 (12), e52030. 10.1371/journal.pone.0052030 23251677 PMC3522645

[B16] Gasteiger E. Hoogland C. Gattiker A. Duvaud S. e. Wilkins M. R. Appel R. D. (2005). “Protein Identification and Analysis Tools on the ExPASy Server,” in The Proteomics Protocols Handbook. Editor Walker J. M. (NewYork: Springer), 571–607. 10.1385/1-59259-890-0:571

[B17] Grennan A. K. (2006). Abiotic stress in rice. An “omic” approach. Plant Physiol. 140 (4), 1139–1141. 10.1104/pp.104.900188 16607027 PMC1435800

[B18] Hadiarto T. Tran L.-S. P. (2011). Progress studies of drought-responsive genes in rice. Plant Cell Rep. 30 (3), 297–310. 10.1007/s00299-010-0956-z 21132431

[B19] Hernandez-Garcia C. M. Finer J. J. (2014). Identification and validation of promoters and *cis*-acting regulatory elements. Plant Sci. 217-218, 109–119. 10.1016/j.plantsci.2013.12.007 24467902

[B20] Hichri I. Barrieu F. Bogs J. Kappel C. Delrot S. Lauvergeat V. (2011). Recent advances in the transcriptional regulation of the flavonoid biosynthetic pathway. J. Exp. Bot. 62, 2465–2483. 10.1093/jxb/erq442 21278228

[B21] Higo K. Ugawa Y. Iwamoto M. Korenaga T. (1999). Plant cis-acting regulatory DNA elements (PLACE) database: 1999. Nucleic Acids Res. 27, 297–300. 10.1093/nar/27.1.297 9847208 PMC148163

[B22] Hirayama T. Shinozaki K. (2010). Research on plant abiotic stress responses in the post-genome era: past, present and future. Plant J. 61 (6), 1041–1052. 10.1111/j.1365-313X.2010.04124.x 20409277

[B23] Horton P. Park K.-J. Obayashi T. Fujita N. Harada H. Adams-Collier C. J. (2007). WoLF PSORT: protein localization predictor. Nucleic Acids Res. 35, W585–W587. 10.1093/nar/gkm259 17517783 PMC1933216

[B24] Hou L. Chen L. Wang J. Xu D. Dai L. Zhang H. (2012). Construction of stress responsive synthetic promoters and analysis of their activity in transgenic *Arabidopsis thaliana* . Plant Mol. Biol. Rep. 30, 1496–1506. 10.1007/s11105-012-0464-0

[B25] Hu B. Jin J. Guo A.-Y. Zhang H. Luo J. Gao G. (2015). GSDS 2.0: an upgraded gene feature visualization server. Bioinformatics 31 (8), 1296–1297. 10.1093/bioinformatics/btu817 25504850 PMC4393523

[B26] International Rice Genome Sequencing Project (2005). The map-based sequence of the rice genome. Nature 436, 793–800. 10.1038/nature03895 16100779

[B27] Jeyasri R. Muthuramalingam P. Satish L. Pandian S. K. Chen J.-T. Ahmar S. (2021). An Overview of Abiotic Stress in Cereal Crops: Negative Impacts, Regulation, Biotechnology and Integrated Omics. Plants 10 (7), 1472. 10.3390/plants10071472 34371676 PMC8309266

[B28] Jin J. Zhang H. Kong L. Gao G. Luo J. (2014). PlantTFDB 3.0: a portal for the functional and evolutionary study of plant transcription factors. Nucl. Acids Res. 42, D1182–D1187. 10.1093/nar/gkt1016 24174544 PMC3965000

[B29] Katiyar A. Smita S. Lenka S. K. Rajwanshi R. Chinnusamy V. Bansal K. C. (2012). Genome-wide classification and expression analysis of MYB transcription factor families in rice and *Arabidopsis* . BMC Genomics 13 (1), 544. 10.1186/1471-2164-13-544 23050870 PMC3542171

[B30] Kayihan D. S. Aksoy E. Kayihan C. (2021). Identification and expression profiling of toxic boron-responsive microRNAs and their targets in sensitive and tolerant wheat cultivars. Turk J Agric 45 (4), 411–433. 10.3906/tar-2102-5

[B31] Komaki S. Sugimoto K. (2012). Control of the plant cell cycle by developmental and environmental cues. Plant Cell Physiology 53 (6), 953–964. 10.1093/pcp/pcs070 22555815

[B32] Krzywinski M. Schein J. Birol İ. Connors J. Gascoyne R. Horsman D. (2009). Circos: an information aesthetic for comparative genomics. Genome Res. 19 (9), 1639–1645. 10.1101/gr.092759.109 19541911 PMC2752132

[B33] Li C. Ng C. K.-Y. Fan L.-M. (2015). MYB transcription factors, active players in abiotic stress signaling. Environ. Exp. Bot. 114, 80–91. 10.1016/j.envexpbot.2014.06.014

[B34] Liang M.-H. Jiang J.-G. (2017). Analysis of carotenogenic genes promoters and WRKY transcription factors in response to salt stress in Dunaliella bardawil. Sci. Rep. 7, 37025. 10.1038/srep37025 28128303 PMC5269594

[B35] Livak K. J. Schmittgen T. D. (2001). Analysis of Relative Gene Expression Data Using Real-Time Quantitative PCR and the 2−ΔΔCT Method. Methods 25, 402–408. 10.1006/meth.2001.1262 11846609

[B36] Mittler R. (2006). Abiotic stress, the field environment and stress combination. Trends Plant Sci. 11 (1), 15–19. 10.1016/j.tplants.2005.11.002 16359910

[B37] Mittler R. Blumwald E. (2010). Genetic engineering for modern agriculture: challenges and perspectives. Annu. Rev. Plant Biol. 61, 443–462. 10.1146/annurev-arplant-042809-112116 20192746

[B38] Muthuramalingam P. Jeyasri R. Bharathi R. K. A. S. Suba V. Pandian S. T. K. Ramesh M. (2020a). Global integrated omics expression analyses of abiotic stress signaling HSF transcription factor genes in *Oryza sativa* L.: An *In Silico* approach. Genomics 112 (1), 908–918. 10.1016/j.ygeno.2019.06.006 31175978

[B39] Muthuramalingam P. Jeyasri R. Selvaraj A. Kalaiyarasi D. Aruni W. Pandian S. T. K. (2021). Global transcriptome analysis of novel stress associated protein (*SAP*) genes expression dynamism of combined abiotic stresses in *Oryza sativa* (L.). J. Biomol. Struct. Dyn. 39 (6), 2106–2117. 10.1080/07391102.2020.1747548 32212961

[B40] Muthuramalingam P. Jeyasri R. Selvaraj A. Pandian S. K. Ramesh M. (2020b). Integrated transcriptomic and metabolomic analyses of glutamine metabolism genes unveil key players in *Oryza sativa* (L.) to ameliorate the unique and combined abiotic stress tolerance. Int. J. Biol. Macromol. 164, 222–231. 10.1016/j.ijbiomac.2020.07.143 32682969

[B41] Muthuramalingam P. Krishnan S. R. Pandian S. Mareeswaran N. Aruni W. Pandian S. K. (2018b). Global analysis of threonine metabolism genes unravel key players in rice to improve the abiotic stress tolerance. Sci. Rep. 8 (1), 1–14. 10.1038/s41598-018-27703-8 29915249 PMC6006157

[B42] Muthuramalingam P. Krishnan S. R. Pothiraj R. Ramesh M. (2017). Global transcriptome analysis of combined abiotic stress signaling genes unravels key players in *Oryza sativa* L.: an *In Silico* approach. Front. Plant Sci. 8, 759. 10.3389/fpls.2017.00759 28555143 PMC5430072

[B43] Muthuramalingam P. Krishnan S. R. Saravanan K. Mareeswaran N. Kumar R. Ramesh M. (2018a). Genome-wide identification of major transcription factor superfamilies in rice identifies key candidates involved in abiotic stress dynamism. J. Plant Biochem. Biotechnol. 27 (3), 300–317. 10.1007/s13562-018-0440-3

[B44] Nakashima K. Yamaguchi-Shinozaki K. Shinozaki K. (2014). The transcriptional regulatory network in the drought response and its crosstalk in abiotic stress responses including drought, cold, and heat. Front. Plant Sci. 5, 170. 10.3389/fpls.2014.00170 24904597 PMC4032904

[B45] Narusaka Y. Nakashima K. Shinwari Z. K. Sakuma Y. Furihata T. Abe H. (2003). Interaction between two cis-acting elements, ABRE and DRE, in ABA-dependent expression of Arabidopsis rd29A gene in response to dehydration and high-salinity stresses. Plant J. 34, 137–148. 10.1104/pp.104.04356210.1046/j.1365-313x.2003.01708.x 12694590

[B46] Noda K.-i. Glover B. J. Linstead P. Martin C. (1994). Flower colour intensity depends on specialized cell shape controlled by a Myb-related transcription factor. Nature 369, 661–664. 10.1038/369661a0 8208293

[B47] Pandey P. Irulappan V. Bagavathiannan M. V. Senthil-Kumar M. (2017). Impact of combined abiotic and biotic stresses on plant growth and avenues for crop improvement by exploiting physio-morphological traits. Front. plant Sci. 8, 537. 10.3389/fpls.2017.00537 28458674 PMC5394115

[B48] Priya P. Jain M. (2013). RiceSRTFDB: a database of rice transcription factors containing comprehensive expression, cis-regulatory element and mutant information to facilitate gene function analysis. Database. 10.1093/database/bat027 PMC3649641 23660286

[B49] Riechmann J. L. Heard J. Martin G. Reuber L. Jiang C.-Z. Keddie J. (2000). Arabidopsis transcription factors: genome-wide comparative analysis among eukaryotes. Science 290 (5499), 2105–2110. 10.1126/science.290.5499.2105 11118137

[B50] Roy S. (2016). Function of MYB domain transcription factors in abiotic stress and epigenetic control of stress response in plant genome. Plant Signal. Behav. 11 (1), e1117723. 10.1080/15592324.2015.1117723 26636625 PMC4871670

[B51] Sadiq Y. Zaid A. Khan M. M. A. (2020). “Adaptive Physiological Responses of Plants under Abiotic Stresses: Role of Phytohormones,” in Plant Ecophysiology and Adaptation under Climate Change: Mechanisms and Perspectives. Editor Hasanuzzaman M., 797–824. 10.1007/978-981-15-2156-0_28

[B52] Sato Y. Antonio B. A. Namiki N. Takehisa H. Minami H. Kamatsuki K. (2010). RiceXPro: a platform for monitoring gene expression in japonica rice grown under natural field conditions. Nucleic Acids Res. 39 (Suppl. l_1), D1141–D1148. 10.1093/nar/gkq1085 21045061 PMC3013682

[B53] Sato Y. Takehisa H. Kamatsuki K. Minami H. Namiki N. Ikawa H. (2013). RiceXPro Version 3.0: Expanding the informatics resource for rice transcriptome. Nucleic Acids Res. 41, D1206–D1213. 10.1093/nar/gks1125 23180765 PMC3531122

[B54] Seo P. J. Lee S. B. Suh M. C. Park M.-J. Go Y. S. Park C.-M. (2011). The MYB96 Transcription Factor Regulates Cuticular Wax Biosynthesis under Drought Conditions inArabidopsis. Plant Cell 23, 1138–1152. 10.1105/tpc.111.083485 21398568 PMC3082259

[B55] Sharoni A. M. Nuruzzaman M. Satoh K. Shimizu T. Kondoh H. Sasaya T. (2011). Gene structures, classification and expression models of the AP2/EREBP transcription factor family in rice. Plant Cell physiology 52 (2), 344–360. 10.1093/pcp/pcq196 21169347

[B56] Singh D. Laxmi A. (2015). Transcriptional regulation of drought response: a tortuous network of transcriptional factors. Front. Plant Sci. 6, 895. 10.3389/fpls.2015.00895 26579147 PMC4625044

[B57] Smita S. Katiyar A. Chinnusamy V. Pandey D. M. Bansal K. C. (2015). Transcriptional regulatory network analysis of MYB transcription factor family genes in rice. Front. plant Sci. 6, 1157. 10.3389/fpls.2015.01157 26734052 PMC4689866

[B58] Szklarczyk D. Morris J. H. Cook H. Kuhn M. Wyder S. Simonovic M. (2017). The STRING database in 2017: quality-controlled protein-protein association networks, made broadly accessible. Nucleic Acids Res. 45, D362–D368. 10.1093/nar/gkw937 27924014 PMC5210637

[B59] Udvardi M. K. Kakar K. Wandrey M. Montanari O. Murray J. Andriankaja A. (2007). Legume transcription factors: global regulators of plant development and response to the environment. Plant Physiol. 144 (2), 538–549. 10.1104/pp.107.098061 17556517 PMC1914172

[B60] Wani S. H. Tripathi P. Zaid A. Challa G. S. Kumar A. Kumar V. (2018a). Transcriptional regulation of osmotic stress tolerance in wheat (*Triticum aestivum* L.). Plant Mol. Biol. 97, 469–487. 10.1007/s11103-018-0761-6 30109563

[B61] Wani W. Masoodi K. Z. Zaid A. Wani S. H. Shah F. Meena V. S. (2018b). Engineering plants for heavy metal stress tolerance. Rend. Fis. Acc. Lincei 29, 709–723. 10.1007/s12210-018-0702-y

[B62] Xie Z. Lee E. Lucas J. R. Morohashi K. Li D. Murray J. A. H. (2010). Regulation of Cell Proliferation in the Stomatal Lineage by theArabidopsisMYB FOUR LIPS via Direct Targeting of Core Cell Cycle Genes. Plant Cell 22 (7), 2306–2321. 10.1105/tpc.110.074609 20675570 PMC2929110

[B63] Yamaguchi-Shinozaki K. Shinozaki K. (2005). Organization of cis-acting regulatory elements in osmotic- and cold-stress-responsive promoters. Trends Plant Sci. 10, 88–94. 10.1016/j.tplants.2004.12.012 15708346

[B64] Yang C. Li D. Liu X. Ji C. Hao L. Zhao X. (2014). OsMYB103L, an R2R3-MYB transcription factor, influences leaf rolling and mechanical strength in rice (Oryza sativaL.). BMC Plant Biol. 14 (1), 158. 10.1186/1471-2229-14-158 24906444 PMC4062502

[B65] Yilmaz A. Nishiyama M. Y. Fuentes B. G. Souza G. M. Janies D. Gray J. (2009). GRASSIUS: A Platform for Comparative Regulatory Genomics across the Grasses. Plant Physiol. 149, 171–180. 10.1104/pp.108.128579 18987217 PMC2613736

[B66] Yu C.-S. Cheng C.-W. Su W.-C. Chang K.-C. Huang S.-W. Hwang J.-K. (2014). CELLO2GO: a web server for protein subcellular localization prediction with functional gene ontology annotation. PLoS One 9, e99368. 10.1371/journal.pone.0099368 24911789 PMC4049835

[B67] Zhang J. Li Y. Jia H.-X. Li J.-B. Huang J. Lu M.-Z. (2015). The heat shock factor gene family in *Salix suchowensis*: a genome-wide survey and expression profiling during development and abiotic stresses. Front. Plant Sci. 6, 748. 10.3389/fpls.2015.00748 26442061 PMC4584977

[B68] Zhang L. Yu S. Zuo K. Luo L. Tang K. (2012). Identification of gene modules associated with drought response in rice by network-based Analysis. PLoS One 7 (5), e33748. 10.1371/journal.pone.0033748 22662107 PMC3360736

[B69] Zhao Y. Xing L. Wang X. Hou Y.-J. Gao J. Wang P. (2014). The ABA receptor PYL8 promotes lateral root growth by enhancing MYB77-dependent transcription of auxin-responsive genes. Sci. Signal. 7 (328), ra53. 10.1126/scisignal.2005051 24894996 PMC4298826

