# Supplementary figures and images for: Global Integrated Genomic and Transcriptomic Analyses of MYB Transcription Factor Superfamily in C3 Model Plant Oryza sativa (L.) Unravel Potential Candidates Involved in Abiotic Stress Signaling
Source: Front Genet. 2022 Jul 8;13:946834. doi: 10.3389/fgene.2022.946834 (PMC9305833; doi:10.3389/fgene.2022.946834)

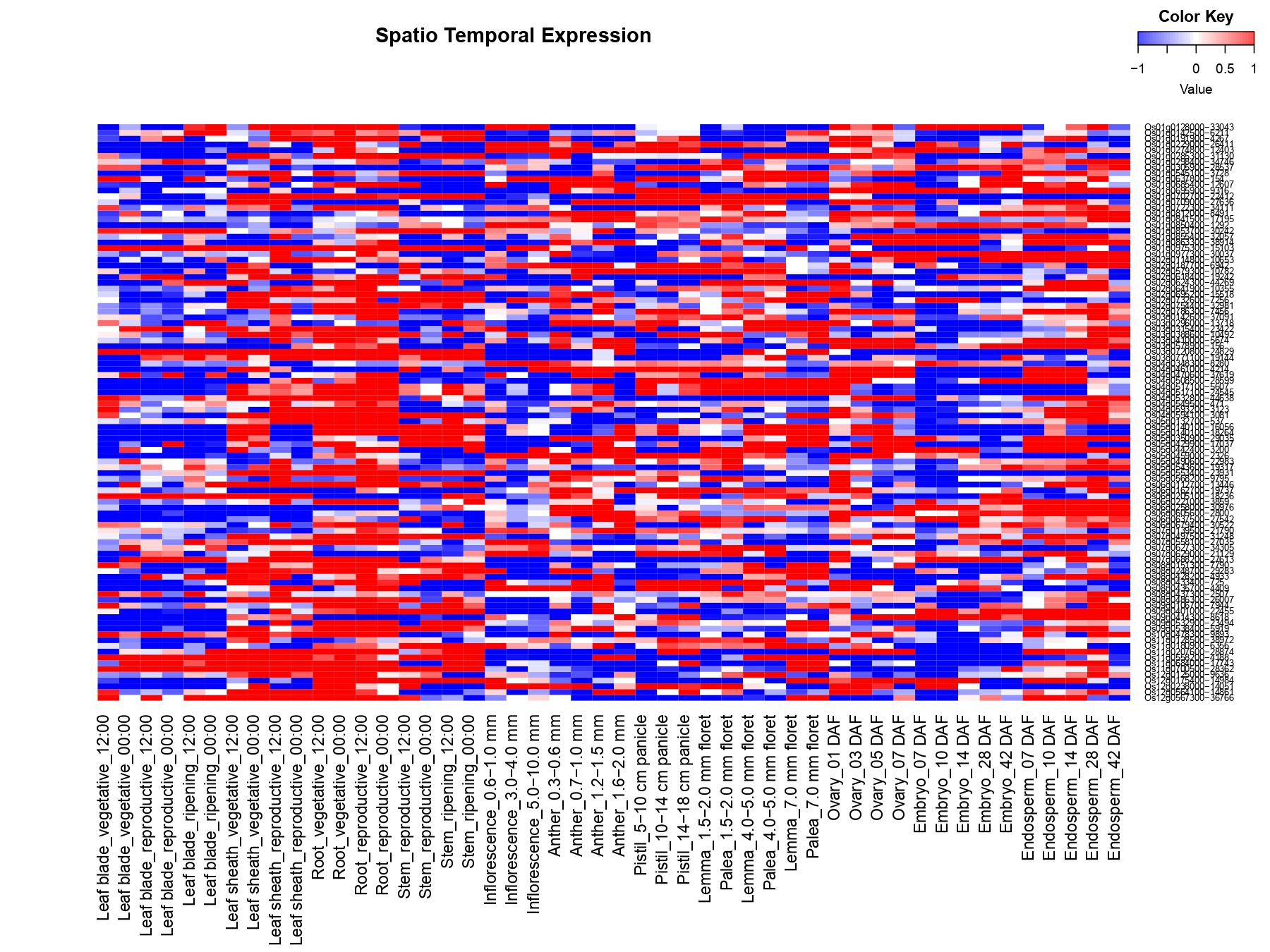

Supplement: Supplementary file 2 [file Image3.JPEG]

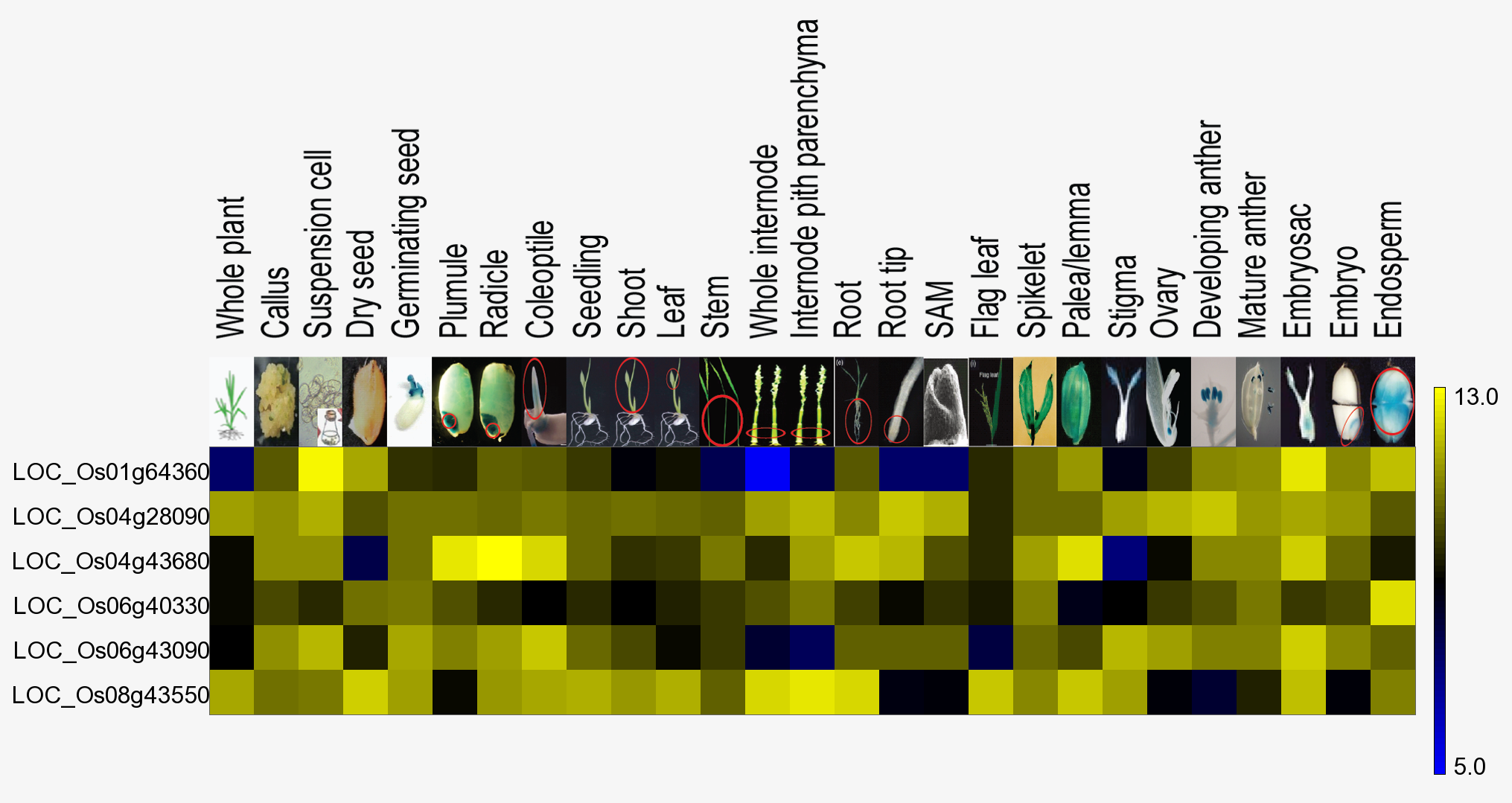

Supplement: Supplementary file 5 [file Image2.PNG]

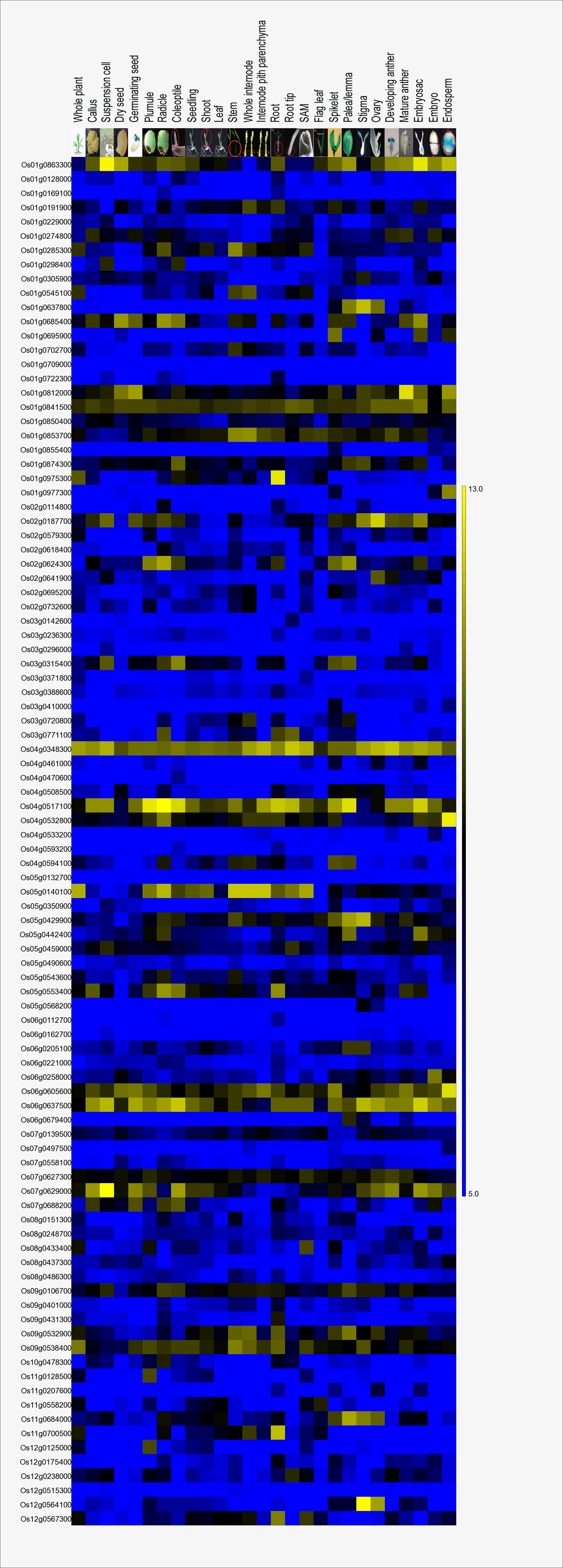

Supplement: Supplementary file 6 [file Image1.PNG]
