# Supplementary material for: Global Integrated Genomic and Transcriptomic Analyses of MYB Transcription Factor Superfamily in C3 Model Plant Oryza sativa (L.) Unravel Potential Candidates Involved in Abiotic Stress Signaling
Source: Front Genet. 2022 Jul 8;13:946834. doi: 10.3389/fgene.2022.946834 (PMC9305833; doi:10.3389/fgene.2022.946834)
Supplement: Supplementary file 3 [file Table3.DOC]

**Supplementary Table S3:** AbSR-*OsMYB* TFs responsible gene orthologous relationships between *O. sativa* and *S. italica*, *S. bicolor*, *Z. mays*

| **S.**  **No** | | **Gene name** | | **Gene ID** | | **Chr. No** | **Start** | **End** | | **Overlapping gene** | **Chr. No** | | **Start** | **End** | **Similarity (%)** |
| --- | --- | --- | --- | --- | --- | --- | --- | --- | --- | --- | --- | --- | --- | --- | --- |
| **MYB (*O. sativa* and *Setaria italica*)** | | | | | | | | | | | | | | | |
| 1 | | *OsMYBR17* | | LOC_Os01g64360 | | 1 | 37357161 | 37358553 | | Si003047m.g | 5 | | 42135061 | 42135267 | 80.7 |
| 2 | | *OsMYB50* | | LOC_Os04g28090 | | 4 | 16407910 | 16415223 | | Si021096m.g | 3 | | 46438190 | 46439134 | 93.3 |
| 3 | | *OsMYB55* | | LOC_Os04g43680 | | 4 | 25657904 | 25659778 | | Si015081m.g | 6 | | 35041101 | 35041448 | 81.6 |
| 4 | | *OsMYB80* | | LOC_Os06g40330 | | 6 | 24004102 | 24011311 | | Si007070m.g | 4 | | 34223083 | 34223331 | 81.9 |
| 5 | | *OsMYB81* | | LOC_Os06g43090 | | 6 | 25898445 | 25899820 | | Si006938m.g | 4 | | 32370385 | 32370729 | 92.2 |
| 6 | | *OsMYB102* | | LOC_Os08g43550 | | 8 | 27545257 | 27546459 | | Si015081m.g | 6 | | 35040996 | 35041448 | 84.1 |
| **MYB (*O. sativa* and *Sorghum bicolor*)** | | | | | | | | | | | | | | | |
| 1 | *OsMYBR17* | | LOC_Os01g64360 | | 1 | | 37357161 | 37358553 | | Sb03g040730 | 3 | | 68257648 | 68257851 | 86.5 |
| 2 | *OsMYB50* | | LOC_Os04g28090 | | 4 | | 16407910 | 16415223 | | Sb03g044450 | 3 | | 71802111 | 71803055 | 93.7 |
| 3 | *OsMYB55* | | LOC_Os04g43680 | | 4 | | 25657904 | 25659778 | | Sb08g001800 | 8 | | 1800642 | 1800989 | 89.8 |
| 4 | *OsMYB80* | | LOC_Os06g40330 | | 6 | | 24004102 | 24011311 | | Sb10g023665 | 10 | | 52338633 | 52338881 | 80.7 |
| 5 | *OsMYB81* | | LOC_Os06g43090 | | 6 | | 25898445 | 25899820 | | Sb10g024950 | 10 | | 54137082 | 54137429 | 90.5 |
| 6 | *OsMYB102* | | LOC_Os08g43550 | | 8 | | 27545257 | 27546459 | | Sb07g024890 | 7 | | 59865046 | 59865312 | 95.5 |
| **MYB (*O. sativa* and *Zea mays*)** | | | | | | | | | | | | | | | |
| 1 | | *OsMYBR17* | | LOC_Os01g64360 | 1 | | 37357161 | 37358553 | Zm00001d012285 | | | 8 | 171872507 | 171872716 | 84.3 |
| 2 | | *OsMYB50* | | LOC_Os04g28090 | 4 | | 16407910 | 16415223 | Zm00001d042287 | | | 3 | 159078156 | 159078968 | 93 |
| 3 | | *OsMYB55* | | LOC_Os04g43680 | 4 | | 25657904 | 25659778 | Zm00001d035918 | | | 6 | 60272476 | 60272808 | 73 |
| 4 | | *OsMYB80* | | LOC_Os06g40330 | 6 | | 24004102 | 24011311 | Zm00001d035918 | | | 6 | 60272482 | 60272790 | 81.2 |
| 5 | | *OsMYB81* | | LOC_Os06g43090 | 6 | | 25898445 | 25899820 | Zm00001d046632 | | | 9 | 99815021 | 99815353 | 95.5 |
| 6 | | *OsMYB102* | | LOC_Os08g43550 | 8 | | 27545257 | 27546459 | Zm00001d035918 | | | 6 | 60272443 | 60272808 | 82.1 |
