# Supplementary material for: Global Integrated Genomic and Transcriptomic Analyses of MYB Transcription Factor Superfamily in C3 Model Plant Oryza sativa (L.) Unravel Potential Candidates Involved in Abiotic Stress Signaling
Source: Front Genet. 2022 Jul 8;13:946834. doi: 10.3389/fgene.2022.946834 (PMC9305833; doi:10.3389/fgene.2022.946834)
Supplement: Supplementary file 4 [file Table1.DOC]

**Supplementary Table xx.** Details of primers used in qRT-PCR analysis.

| **Gene** | **Forward primer (5'-3')** | **Reverse primer (5'-3')** |
| --- | --- | --- |
| *OsMYBR17* | GGAGGACAAGGTGTTCGAGA | CGTTGCCGTCGTTATCGT |
| *OsMYB50* | TCTCAGTGCCTTGAGCGTTA | CCCTTGCCTCAGAAAGCATC |
| *OsMYB55* | GAAGCACAAGAAGCCGAAGA | GAAGGAGCTCTCCTCCTTGG |
| *OsMYB80* | ACGTGCCAAGAAACGAACAA | GTGGCAATGGCACTACCAAA |
| *OsMYB81* | CTTCCAAGAACTCCCGTCCT | GCCATGTACTTGTGCACCTC |
| *OsMYB102* | TCCGCTGGATCAACTACCTC | TTGATCTCGTTGTCCGTCCT |
| *Actin2* | CGCATATGTGGCTCTTGACT | GGGCACCTAAATCTCTCTGC |
